# Reducing Amyloid Plaque Burden via Ex Vivo Gene Delivery of an Aβ-Degrading Protease: A Novel Therapeutic Approach to Alzheimer Disease

**DOI:** 10.1371/journal.pmed.0040262

**Published:** 2007-08-28

**Authors:** Matthew L Hemming, Michaela Patterson, Casper Reske-Nielsen, Ling Lin, Ole Isacson, Dennis J Selkoe

**Affiliations:** 1 Center for Neurologic Diseases, Brigham and Women's Hospital and Harvard Medical School, Boston, Massachusetts, United States of America; 2 Neuroregeneration Laboratories, McLean Hospital and Harvard University Udall Parkinson's Disease Research Center of Excellence, Belmont, Massachusetts, United States of America; Farber Institute, United States of America

## Abstract

**Background:**

Understanding the mechanisms of amyloid-β protein (Aβ) production and clearance in the brain has been essential to elucidating the etiology of Alzheimer disease (AD). Chronically decreasing brain Aβ levels is an emerging therapeutic approach for AD, but no such disease-modifying agents have achieved clinical validation. Certain proteases are responsible for the catabolism of brain Aβ in vivo, and some experimental evidence suggests they could be used as therapeutic tools to reduce Aβ levels in AD. The objective of this study was to determine if enhancing the clearance of Aβ in the brain by ex vivo gene delivery of an Aβ-degrading protease can reduce amyloid plaque burden.

**Methods and Findings:**

We generated a secreted form of the Aβ-degrading protease neprilysin, which significantly lowers the levels of naturally secreted Aβ in cell culture. We then used an ex vivo gene delivery approach utilizing primary fibroblasts to introduce this soluble protease into the brains of β-amyloid precursor protein (APP) transgenic mice with advanced plaque deposition. Brain examination after cell implantation revealed robust clearance of plaques at the site of engraftment (72% reduction, *p* = 0.0269), as well as significant reductions in plaque burden in both the medial and lateral hippocampus distal to the implantation site (34% reduction, *p* = 0.0020; and 55% reduction, *p* = 0.0081, respectively).

**Conclusions:**

Ex vivo gene delivery of an Aβ-degrading protease reduces amyloid plaque burden in transgenic mice expressing human APP. These results support the use of Aβ-degrading proteases as a means to therapeutically lower Aβ levels and encourage further exploration of ex vivo gene delivery for the treatment of Alzheimer disease.

## Abstract 2

Matthew Hemming and colleagues describe the ex vivo gene delivery of an Aβ-degrading protease that reduces amyloid plaque burden in transgenic mice expressing human amyloid precursor protein.

## Editors' Summary

**Background.:**

Alzheimer disease is the most common form of dementia and is increasingly common as people age. It may affect up to half of the population in people over 85 years of age. The classic pathological features of Alzheimer disease in the brain were described 100 years ago by a German neuropathologist, Alois Alzheimer. They consist of plaques, which are made up of a protein called amyloid-β protein (shortened to Aβ); and tangles of another protein, called tau. These proteins accumulate especially in areas of the brain where memory and thought are processed and are believed to be toxic to neurons. There are a number of inherited forms of Alzheimer disease which are caused by mutations either in the protein from which Aβ is derived, called beta-amyloid precursor protein (shortened to APP) or in other proteins that act to release the Aβ protein from APP. Research on these inherited forms of Alzheimer disease has helped in the understanding of how plaques accumulate, which has subsequently led to new potential approaches to the treatment of Alzheimer disease such as lowering the production of Aβ from APP or enhancing clearance of the plaques.

**Why Was This Study Done?:**

The researchers here wanted to investigate the use of a type of gene therapy called ex vivo (which means out of body) gene therapy, in which cells are taken from an individual, genetically altered, then put back into the individual they were taken from. This approach has already been studied in some human diseases and conditions including hemophilia, cancer, and spinal cord injury. The researchers here wanted to investigate whether they could use this approach to deliver to the brain one of the proteases that breaks down amyloid plaques.

**What Did the Researchers Do and Find?:**

The researchers used a mouse model of Alzheimer disease in which, as the mice age, they develop plaques in the brain made of the same protein found in human Alzheimer disease. The researchers took a particular type of cell from the mice called a fibroblast, used a virus called a lentivirus to insert into the cells a protease called neprilysin that can degrade Aβ, and then injected these altered cells into the brains of the mice. They then compared results of these experiments with what happened when cells with a control (an inactive virus) were injected. They found that when the active protease was put into the brains of mice there was a substantial clearing of plaques especially in the areas close to the injection site, compared with the control mice.

**What Do These Findings Mean?:**

These results suggest that this ex vivo gene therapy approach to Alzheimer disease is worth considering further. However, despite showing that the plaques were cleared the researchers did not show whether or not there was any effect on the behavior of the mice, i.e., whether there was any effect on the symptoms that the plaques cause. In addition, before this approach could be used in humans more work would need to be done, including showing that the lentivirus and the protease used were safe in humans, and the injection could be scaled up to the much larger human brain.

**Additional Information.:**

Please access these Web sites via the online version of this summary at http://dx.doi.org/10.1371/journal.pmed.0040262.

- The slides from the original cases that Alois Alzheimer described are available online
- Current research is discussed by the Alzheimer Research Forum
- News and information for researchers, doctors, and patients, including a 24-hour help line, are available from the Alzheimer's Association
- Medline Plus, the health information site for patients from the US National Library of Medicine, has a page of links on Alzheimer disease
- The US National Institutes of Health National Institute of Aging has a fact sheet on Alzheimer disease, which is also available in Spanish
- The UK's National Health Service online information site has information on Alzheimer disease

## Introduction

The pathologic hallmarks of Alzheimer disease (AD) are extracellular plaques of amyloid-β protein (Aβ) and intraneuronal neurofibrillary tangles of tau protein, both of which accumulate in brain regions mediating memory and cognition [1]. All known early-onset, inherited forms of AD arise from mutations in the β-amyloid precursor protein (APP), from which the Aβ peptide is generated, or the presenilins, which are the proteases that effect the cleavage of APP, which liberates Aβ. Both amyloid plaques and soluble Aβ oligomers are believed to have neurotoxic effects [2,3], and evidence suggests that Aβ neurotoxicity can promote neurofibrillary tangle formation [4,5]. Though approved treatments for AD ameliorate only the symptoms, potentially disease-modifying interventions under development focus on therapeutically lowering Aβ production [6] or enhancing Aβ clearance [7,8].

The role of Aβ degradation in the clearance of the Aβ peptide is becoming more broadly understood and appreciated, with the proteases neprilysin [9], insulin-degrading enzyme [10,11], endothelin-converting enzymes 1 and 2 [12], plasmin [13], and cathepsin B [14] all capable of regulating Aβ levels in vivo. Supporting a therapeutic function for Aβ-degrading proteases, both transgenic overexpression [15] and direct viral vector injection [14,16,17] of these enzymes have been shown to potently lower Aβ levels and plaque burden and reduce Aβ-associated neuropathology. The applicability of these approaches for AD treatment in humans is uncertain, and alternative methods of gene delivery merit exploration.

In ex vivo gene therapy, cells are taken from a patient, genetically modified in vitro, then implanted back into the patient to exert their new salutary effects. This approach has produced therapeutic improvements in experimental models of human diseases and conditions including hemophilia [18], retinal degeneration [19], cancer [20], spinal cord injury [21], myopathy [22], ischemia [23], Parkinson disease [24], Huntington disease [25], amyotrophic lateral sclerosis [26], and Alzheimer disease [27]. Several of these potential treatments have advanced to human trials [28–31], with encouraging outcomes for patients. In a recent Phase I clinical trial, autologous fibroblasts from AD patients were modified to produce nerve growth factor and implanted into the forebrain, where they enhanced cholinergic function [28]. These efforts demonstrate the feasibility of ameliorating cholinergic cell death by providing trophic support using an ex vivo gene delivery approach. However, because Aβ-mediated synaptic function and cytotoxicity are likely to be upstream of neurotransmitter abnormalities in AD, enhancing Aβ clearance represents a more attractive target for gene therapy in this disorder.

Neprilysin (NEP) is a type II transmembrane protein bound to the cell membrane, where it normally degrades Aβ intracellularly and on the cell surface [32]. Cerebral NEP levels have been reported to decrease with age and in AD [33,34], which may contribute to disease pathogenesis by compromising Aβ catabolism. As an approach to elevate NEP levels and improve the enzyme's access to extracellular Aβ, we replaced the transmembrane domain of NEP with a signal peptide, which produced a soluble and secreted form of NEP, termed sNEP. We then used ex vivo gene delivery utilizing syngenic primary fibroblasts to introduce sNEP into the brains of APP transgenic mice that had advanced plaque deposition and studied the effect of this protein on brain amyloid pathology.

## Materials and Methods

### sNEP Cloning

The sNEP construct was generated by an overlapping PCR method to fuse the 29 amino-acid signal peptide of angiotensin-converting enzyme (ACE) to luminal residues 52–750 of neprilysin. Two PCR amplicons were generated that consisted of the ACE signal peptide and the NEP luminal residues, both with a complimentary 23-basepair overlap added to the primer. In a third PCR reaction, the overlapping amplicons were fused using the ACE signal peptide 5′ and NEP 3′ primers to produce the cDNA encoding sNEP. All constructs were cloned into the pcDNA3.1/Hygro vector (Invitrogen, http://www.invitrogen.com/) for transient and stable expression. The human ACE and human NEP cDNAs have been previously described [15,35]

### Cell Culture and Lentiviral Transduction

Chinese hamster ovary (CHO) and human embryonic kidney (HEK) 293 cells were grown in Dulbecco's modified Eagle's medium containing 10% fetal bovine serum, 2 mM L-glutamine, 100 μg/ml penicillin, and 100 μg/ml streptomycin. HEK cells stably expressing APP_695_ bearing the K595N/M596L AD-causing mutation [36] and CHO cells stably transfected with APP_751_ with the V717F AD-causing mutation [37] were maintained in medium containing 200 μg/ml G418. Transfections were performed with Fugene 6 (Roche, http://www.roche.com/), and stable cell lines were selected using 400 μg/ml hygromycin B. Cells were conditioned for Aβ measurements and protein analysis in Opti-MEM I (Invitrogen) for 16–18 h. For cell-free Aβ degradation assays, conditioned medium from APP-overexpressing CHO cells was combined with 10-fold concentrated conditioned medium from CHO cells overexpressing empty vector, NEP, or sNEP and incubated at 37 °C for 18 h.

Lentiviral vectors were generated by inserting the sNEP and GFP cDNAs into pCDH1 (System Biosciences, http://www.systembio.com/) and were cotransfected with the helper plasmids delta8.9 and VSV-G as previously described [38] into 293-FT cells (Invitrogen). Conditioned media were collected 50–60 h later, centrifuged at 500 *g* for 5 min to remove suspended cells, and stored at −80 °C. Viral multiplicity of infection was estimated by cellular fluorescence and Western blotting by transducing 293-FT cells with serial dilutions of viral supernatant. Cells were transduced for experiments at a multiplicity of infection of six to ten viral particles per cell with media containing 6 μg/ml polybrene.

### Immunoblotting

Cells and tissue were lysed in 50 mM Tris-HCl (pH 7.4), 150 mM NaCl, 1% NP-40, protease inhibitor cocktail (Roche), 2 mM 1,10-phenanthroline, and 5 mM EDTA, and the extracts centrifuged at 1,000 *g* for 10 min to remove nuclei. Protein concentrations were determined using a bicinchoninic acid-based assay (Pierce, http://www.perbiodirect.com/). Samples were then subjected to SDS-PAGE and Western blotting. We detected NEP with monoclonal antibody 56C6 (1:100 dilution; Abcam, http://www.abcam.com/); GFP with polyclonal antibody A11122 (1:1,000 dilution; Invitrogen); Aβ with monoclonal antibody 6E10 (1:1,000 dilution; Abcam); and APP with polyclonal antibody C9 (1:1,000 dilution) [35]. Deglycosylation was performed using PNGase F to remove N-linked sugars (Prozyme, http://www.prozyme.com/). Western blots were probed with anti-mouse or anti-rabbit secondary antibodies (1:10,000 dilution) conjugated to Alexa Fluor 680 (Molecular Probes, http://probes.invitrogen.com/) or IRdye 800 (Rockland Immunochemicals, http://www.rockland-inc.com/). Blots were detected using the Odyssey infrared imaging system (LI-COR, http://www.licor.com/).

### NEP Activity Assay

NEP proteolytic activity was measured using the substrate 3-dansyl-D-Ala-Gly-p-(nitro)-Phe-Gly (DAGNPG; Sigma, http://www.sigmaco.com.au/) [39,40]. Cell lysate (100 μg) or the same relative amount of concentrated conditioned medium (prepared without protease inhibitors) was incubated with 50 μM DAGNPG and 1 μM captopril (to inhibit any ACE cleavage of DAGNPG) in a volume of 200 μl at 37 °C. Reactions were stopped by heating samples to 100 °C for 5 min, then centrifuging at 5,000 g for 5 min to remove the denatured protein. The whole supernatant was diluted into 400 μl of 50 mM Tris (pH 7.4) and fluorescence determined using a Victor2 multilabel plate reader (excitation 342 nm; emission 562 nm).

### ELISA

ELISAs for Aβ were performed as previously described [39,41] with few modifications. 96-well ELISA plates (Costar, http://www.corning.com/) were coated with 3.5 μg/ml of the capture antibody. Aβ was measured by capturing with monoclonal antibody 266, specific to residues 13–28 of Aβ. Captured Aβ was detected with biotinylated monoclonal antibody 3D6, specific to residues 1–5 of the Aβ N terminus (all ELISA antibodies were gifts of Elan Pharmaceuticals, http://www.elan.com/). ELISAs were developed by incubating the Aβ-bound biotinylated 3D6 with avidin-horseradish peroxidase (Vector Labs, http://www.vectorlabs.com/) followed by a 1 h incubation with QuantaBlu fluorogenic peroxidase substrate (Pierce), and the resulting fluorescence (excitation 340 nm; emission 400 nm) was measured. Plate washing after the antibody and enzyme binding steps was performed three times for 1 min with Tris-buffered saline, 0.05% Tween 20.

### Primary Fibroblast Generation and Transduction

Primary fibroblasts were generated from young wild-type littermates of J20 transgenic mice that expressed human APP. Skin biopsies were swabbed with 70% ethanol to sterilize the tissue, and were then minced with sterile scalpel blades in 0.25% trypsin-EDTA. Minced tissue was incubated in trypsin-EDTA at 37 °C for 15 min and triturated to further disrupt the tissue. Cells remaining in suspension were plated in growth medium and allowed to expand to 90% confluency. Fibroblasts were then passaged and transduced with two serial applications of either sNEP (treatment cells) or GFP (control cells) lentiviral vectors to attain strong transgene expression in all cells. Fibroblasts were passaged a maximum of five times before injections. Confluent cultures of transduced fibroblasts were maintained to observe cell longevity and transgene expression. After 12 mo in culture, the cells remained metabolically active and continued to express the introduced transgene.

### Mice and Surgical Procedures

The J20 line of APP_Swe/Ind_ heterozygous transgenic mice (C57BL/6 × DBA2 background) [42] were aged to approximately 22–23 mo before surgery to allow for substantial plaque deposition. For surgical procedures, mice received intraperitoneal injections of a preanesthetic mixture (a 1:1 solution of atropine sulfate and acepromazine maleate), followed by a 2:1 solution of ketamine and xylazine. Mice received a unilateral hippocampal injection of 500,000 fibroblasts expressing sNEP or GFP to allow for comparison to the contralateral side. The stereotaxic coordinates for graft placement were calculated from bregma: AP −3.3 mm; L −3.3 mm; DV −3.12 mm and −1.9 mm. Cells, in a total volume of 4 μl, were injected at two dorsoventral positions to better distribute the graft. Brains were harvested 28 d after surgery and fixed in 10% formalin for subsequent immunohistochemical analysis. All animal procedures were approved by the Harvard Standing Committee for Animal Use and by the Animal Care and Use Committee at McLean Hospital (Belmont, Massachusetts, United States).

### Immunohistochemistry and Image Analysis

Sagittal brain sections of 18 μm thickness were deparaffinized and hydrated through a series of graded alcohol steps and washed in phosphate-buffered saline. Endogenous peroxidase activity was quenched with 0.6% hydrogen peroxide in methanol for 15 min. Sections were blocked in serum for 25 min, then incubated with the anti-Aβ antibody R1282 (1:1,000 dilution) [39], anti-NEP antibody 56C6 (1:100), anti-GFP antibody A11122 (1:100), or anti-glial fibrillary acidic protein (GFAP) antibody (1:1,000; Dako, http://www.dako.com/) for 2 h. After washing, an anti-rabbit or anti-mouse biotinylated secondary antibody was applied for 30 min, washed, and developed using the avidin/biotin/HRP method (ABC Elite Kit, Vector Labs) and DAB chromogenic reaction (Liquid DAB, BioGenex, http://www.biogenex.com/). Images were captured and quantified from approximately 20 stained sections per brain of each mouse using a 2× or 5× objective. Thioflavin-S staining was performed by incubating tissue sections for 8 min in 1% thioflavin-S, followed by washing in 80% and 95% ethanol, then water. The brain area covered by Aβ, thioflavin plaques, and GFAP staining was determined in a blinded fashion using IPLab Spectrum 3.1 Image Analyzer software (Signal Analytics, http://www.scanalytics.com/) and NIH ImageJ software [43]. The ratio between the immunohistochemical and thioflavin staining on the ipsilateral versus contralateral hemispheres was calculated for comparisons.

### Statistical Analysis

The data were analyzed using a one-way analysis of variance and Tukey post-hoc comparison or a two-tailed Student *t*-test, where appropriate. Calculated comparisons of *p* < 0.05 were considered significant. All reported values represent the means ± standard error of the mean (SEM).

## Results

### NEP Expression and Generation of sNEP

**Figure 1:**
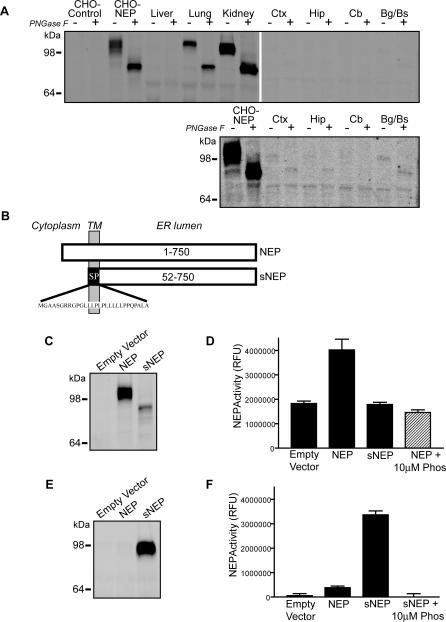
Tissue Distribution of NEP and Generation of Secreted NEP (A) Identical amounts of protein from transfected CHO cells and the indicated mouse tissues were probed by NEP immunoblot. CHO cells were transiently transfected with empty vector (CHO-control) or human NEP (CHO-NEP). The indicated peripheral tissues and brain regions (Bg/Bs, basal ganglia/brainstem; Cb, cerebellum; Ctx, cortex; Hip, hippocampus) were homogenized and probed by Western blot for NEP. The lower blot is a longer exposure of the brain samples, demonstrating low levels of NEP in the brain. Each sample was either left untreated (−) or deglycosylated with PNGase F (+) to remove N-linked sugars. (B) Schematic representation of wild-type NEP and sNEP proteins. The sNEP construct was generated by replacing the NEP transmembrane (TM) domain and cytosolic N terminus with a signal peptide (SP) ending at the luminal residue 52 of NEP. Cleavage of the signal peptide produces a soluble, secreted form of NEP. (C) NEP immunoblot of cellular lysates from CHO cells transfected with empty vector, NEP, or sNEP constructs. (D) NEP activity assay in which the fluorogenic NEP substrate DAGNPG was incubated with lysates from CHO cells stably transfected with the indicated constructs. NEP enzymatic activity was inhibited by phosphoramidon. (E) NEP immunoblot of conditioned medium from CHO cells transfected with the indicated constructs. (F) NEP activity assay on conditioned media from cells transfected with the indicated constructs. NEP activity assays of conditioned media (F) were performed using the same relative amounts of material as for the lysates (D). Immunoblots are representative of at least three experiments; NEP activity assays report the mean ± SEM of six experiments.

Several studies point to NEP as one of the prominent Aβ-degrading proteases within the brain [9,15,44,45]. To determine the tissue distribution of NEP in the brain and several peripheral tissues, we dissected cortex, hippocampus, cerebellum, and the basal ganglia/brainstem from the murine brain and compared protein expression levels to those in the liver, lung, and kidney. As positive and negative controls, lysates from CHO cells transfected with NEP or empty vector control were examined in parallel. Equal amounts of protein from each sample were examined by quantitative fluorescent Western blotting. Because transfected NEP may run as a broad band due to its varying degrees of glycosylation, all samples were assessed in the absence or presence of PNGase F to remove N-linked glycosylation and reduce NEP immunoreactivity to a single signal. Mouse lung and kidney express high levels of NEP that comigrates with transfected human NEP, whereas the liver lacks NEP expression (Figure 1A). In contrast to lung and kidney, brain levels of NEP are very low and detectable only upon longer blot exposure (Figure 1A, lower blot). Thus, although NEP has been characterized as an essential protease for Aβ catabolism, its protein expression levels are relatively low in the brain compared to peripheral tissues that express NEP. Moreover, since cerebral NEP expression decreases with age and in AD [33,34], elevating NEP levels could be neuroprotective by enhancing Aβ catabolism.

In exploring potential therapeutic approaches to reducing brain Aβ levels by elevating NEP expression, we evaluated a secreted form of NEP, sNEP. A secreted Aβ-degrading protease would possess the advantage of diffusion through the interstitial fluid, where it may clear Aβ at sites of extracellular deposition. To generate this sNEP construct, we used an overlapping PCR method to fuse the 29-amino acid signal peptide from ACE to the luminal domain of NEP beginning at residue 52 (Figure 1B). This construct, upon being translated and inserted into the endoplasmic reticulum membrane, has the signal peptide removed to liberate a soluble, secreted form of NEP.

Transfecting cells with empty vector, NEP, or the sNEP constructs and examining cell lysates by Western blotting revealed a robust immunoreactive NEP band in the wild-type NEP condition and a weaker, incompletely glycosylated, lower molecular weight signal in the sNEP lane (Figure 1C). Measuring NEP enzymatic activity in cell lysates demonstrated NEP activity only in the wild-type NEP condition, and this was inhibitable to baseline signal by the metalloprotease inhibitor phosphoramidon (Figure 1D). The incompletely glycosylated intracellular sNEP showed no enzymatic activity, consistent with reports that glycosylation is required for NEP activity [46]. Concentrating the conditioned media from these cells for Western blot analysis revealed a strong signal for sNEP, consistent with proper sNEP expression and secretion (Figure 1E). Quantifying NEP activity in conditioned media (using the same relative amount of material used for cell lysates in Figure 1D) demonstrated robust NEP activity in the sNEP samples alone, and this was again completely inhibited by phosphoramidon (Figure 1F). These data demonstrate that the sNEP construct is processed and secreted by cells as expected and that sNEP is enzymatically active against a prototypical NEP substrate.

### sNEP Reduces Aβ Levels in Cell Culture

**Figure 2:**
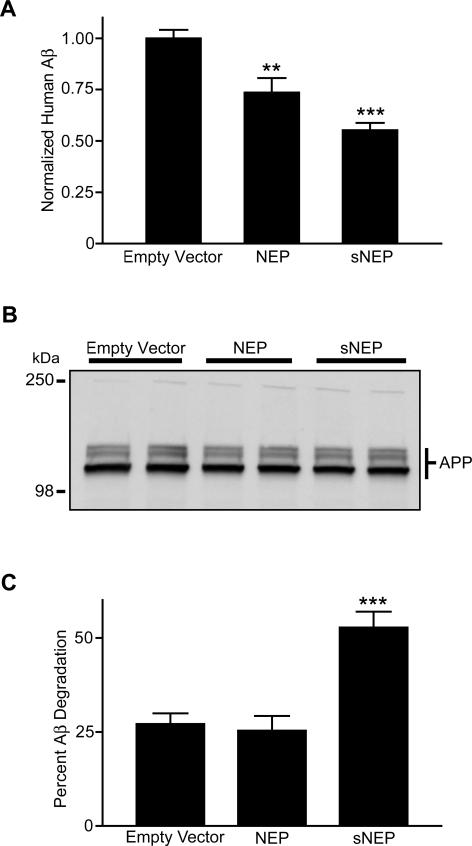
Clearance of the Aβ Peptide by NEP and sNEP (A) CHO cells overexpressing APP were cocultured with CHO cells stably expressing either empty vector, NEP, or sNEP constructs. Equal numbers of cells were seeded, grown to confluence, and media were conditioned for 18 h. Conditioned media were analyzed for total Aβ levels by ELISA. (B) Cellular lysates from the coculture in (A) were probed by Western blotting for APP (which presents in both mature and immature glycosylated forms), demonstrating equal amounts of APP expression. Each condition is shown in duplicate. (C) Conditioned media from CHO cells stably transfected with APP were incubated in vitro with conditioned media from CHO cells expressing empty vector, NEP, or sNEP. The conditioned media were combined and incubated at 37 °C for 18 h. Aβ levels were determined by ELISA. The immunoblot for APP (B) is representative of four experiments; ELISA values for Aβ represent the mean ± SEM of five experiments. For comparisons to the empty vector condition, ** *p* < 0.01 and *** *p* < 0.001.

To determine if the sNEP construct is able to degrade naturally secreted Aβ to a similar extent as wild-type NEP, we cocultured CHO cells stably expressing APP at a 1:1 ratio with CHO cells expressing either empty vector as control, wild-type NEP, or sNEP. Cells were allowed to grow to confluency, and media were conditioned for Aβ ELISA determinations. Both wild-type NEP and sNEP were capable of promoting Aβ catabolism, with the NEP and sNEP conditions reducing Aβ levels in the media to 74% and 55% of control, respectively (*p* < 0.01 for NEP and *p* < 0.001 for sNEP) (Figure 2A). These reductions in Aβ levels were not due to differences in the proliferation rates of the cell lines, as there was equivalent APP expression in each coculture condition (Figure 2B), indicating a similar number of APP expressing cells at confluency.

In order to clarify the location of NEP- and sNEP-mediated Aβ degradation, we mixed conditioned media from the APP-overexpressing cell line with concentrated media from cells expressing empty vector, wild-type NEP, or sNEP. Only the sNEP-conditioned media exhibited an increased capacity to degrade Aβ compared to control (*p* < 0.001) (Figure 2C), elevating the amount of Aβ degraded 2-fold. Taken together, these data demonstrate that wild-type NEP degrades Aβ principally while anchored to the cell membrane, whereas sNEP is efficiently secreted into the extracellular environment to mediate Aβ catabolism.

### Generation of Viral and Cell-Based Aβ-Degrading Vectors

**Figure 3:**
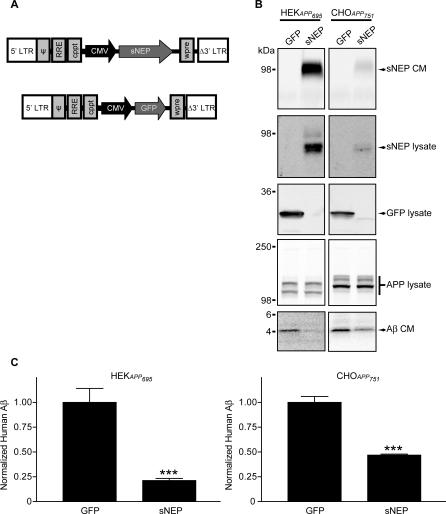
Characterization of a sNEP Lentiviral Construct (A) Schematic representation of the lentiviral constructs expressing sNEP and GFP. Blocks indicate lentiviral genetic components. Internal promoters and transgenes are indicated by arrows. (B) HEK cells stably transfected with APP_695_ K595N/M596L (HEK*APP_695_*) and CHO cells stably transfected with APP_751_ V717F (CHO*APP_751_*) were transduced with the lentiviral constructs in (A). Lysates and conditioned media (CM) were collected and probed for sNEP, GFP, APP, and Aβ. (C) Media from the lentivirally transduced cells were conditioned, and Aβ levels were determined by ELISA. Immunoblots are representative of four experiments; Aβ ELISAs represent the mean ± SEM of four experiments, compared to GFP transduced cells: *** *p* < 0.001

To pursue an ex vivo gene delivery approach, we generated lentiviral vectors able to efficiently transduce most cell types. Retroviral vectors have been routinely utilized to genetically enhance cells ex vivo prior to engraftment for the experimental treatment of human diseases [47–49]. We inserted the cDNA of sNEP or GFP (as control) into a lentiviral vector driven by the cytomegalovirus promotor (Figure 3A). The resulting vectors were able to transduce all cultured cell types tested (HeLa, SH-SY5Y, SK-N-SH, HEK, CHO, and primary and immortalized fibroblasts), and produced high levels of GFP or sNEP expression. To assess the ability of the sNEP lentiviral vector to reduce Aβ levels in APP overexpressing cell lines, we transduced HEK cells stably expressing a familial AD mutant form of APP_695_ and CHO cells stably expressing a familial AD mutant form of APP_751_ with the sNEP or GFP vectors. Examining cell lysates and conditioned media by Western blot showed strong transgene expression, with HEK cells attaining higher sNEP and GFP production than did CHO cells (Figure 3B, top three blot pairs). Expression of sNEP did not change APP levels in these cells compared with GFP transduction (Figure 3B, fourth blot pair); however, sNEP expression resulted in a marked decrease in Aβ monomer in the conditioned media (Figure 3B, fifth blot pair). Quantifying Aβ levels in the conditioned media by ELISA demonstrated significant reductions in Aβ due to sNEP expression in both HEK and CHO APP-overexpressing cell lines (*p* < 0.001) (Figure 3C).

**Figure 4:**
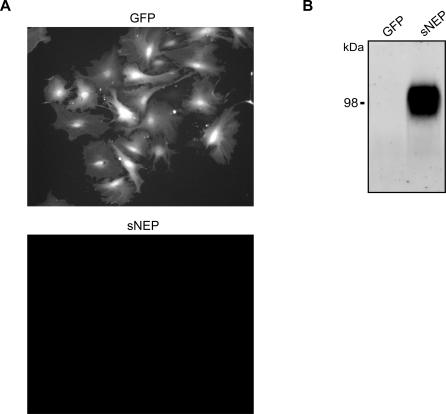
Generation of Primary Mouse Fibroblasts Expressing GFP and sNEP Fibroblast cultures were generated from wild-type littermates of J20 APP transgenic mice. (A) Live-cell fluorescent imaging of primary fibroblasts transduced with lentiviral vectors expressing GFP (top) or sNEP (bottom). (B) Conditioned media from the primary fibroblasts were probed for the presence of sNEP. The immunoblot is representative of three experiments.

Fibroblasts were chosen as the optimal cell type for use in ex vivo gene delivery for a number of reasons. Fibroblasts survive grafting into several organs, including the brain; they survive and can sustain transgene expression for years [28]; they do not form tumors or migrate from the graft site [47]; they cause no detectible toxicity to the host tissue [50]; and they can be noninvasively extracted from patients via skin biopsy. For this study, fibroblasts were prepared from the wild-type littermates of the J20 APP transgenic mice. Cells were dissociated from skin biopsies and plated in growth medium for brief expansion in culture. Following their first passage in culture, the cells were transduced with either the sNEP or the GFP lentiviral vector. The fibroblasts transduced with GFP produce a strong native fluorescent signal (Figure 4A), while cells transduced with sNEP robustly secrete the sNEP protein into the extracellular medium (Figure 4B). To minimize the possibility of in vitro cell transformation, the fibroblasts used in the study were not passaged more the five times before engraftment into the experimental transgenic mice.

### sNEP Cell Grafting Reduces Plaque Burden Locally and Distal to the Implantation Site

**Figure 5:**
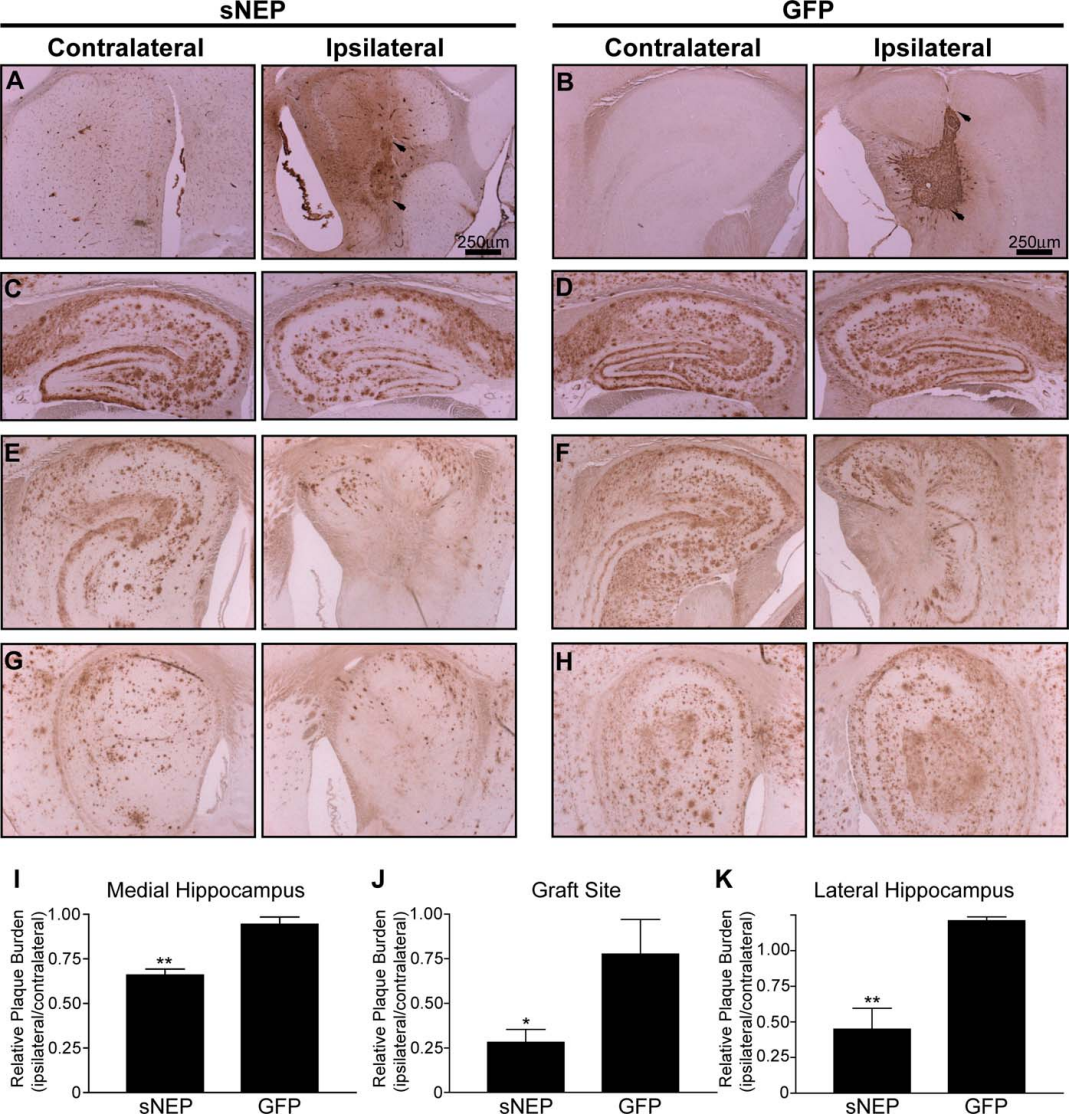
Reduction in Hippocampal Plaque Burden following sNEP Cell Engraftment Aged J20 APP transgenic mice were implanted with fibroblasts expressing either sNEP (*n* = 5) (A, C, E, and G) or GFP (*n* = 3) (B, D, F, and H). Cells were stereotaxically placed into the right (ipsilateral, right) hippocampus, with the uninjected left (contralateral, left) hippocampus serving as control. Brains were harvested for analysis 28 d after surgery. Grafted cells were immunoreactive for either NEP (A) or GFP (B), with superior and inferior aspects of the graft, respectively, indicated by arrows. Images (C–H) show staining for plaque burden at sites medial to the graft (C and D), at the site of the graft (E and F), and at sites lateral to the graft (G and H). These fields were quantified by calculating the ratio of the area covered by plaques on the ipsilateral versus the contralateral side; quantification was done in a blinded fashion. This ratio was determined separately for the sNEP and GFP groups and compared at the hippocampus medial to the graft (I), the graft site (J), and the hippocampus lateral to the graft (K). Data represent the ratio's mean ± SEM, compared to GFP control: ** *p* = 0.0020 for the medial hippocampus (I); * *p* = 0.0269 for the graft site (J); and ** *p* = 0.0081 for the lateral hippocampus (K).

A cohort of J20 APP transgenic mice was aged to achieve significant plaque deposition [42] for use in these studies. To each of these mice, 500,000 cells expressing sNEP or GFP were stereotaxically injected unilaterally into the hippocampus (stereotaxic coordinates: AP −3.3 mm; L −3.3 mm; DV −3.12 and −1.9 mm), with the uninjected contralateral hippocampus serving as a control. Cells were injected at two dorsoventral positions to better distribute the graft. The brains of the mice were harvested and immunohistochemically assayed 28 d after surgery. Mice implanted with sNEP grafts exhibited NEP immunoreactivity at the graft site, as well as some diffuse NEP signal in nearby areas of the hippocampus that extended approximately 800 μm from the grafted cells (Figure 5A, right photomicrograph), consistent with sNEP secretion. Endogenous NEP immunoreactivity is localized to the cerebral blood vessels and the choroid plexus (Figure 5A, left photomicrograph). GFP fibroblasts exhibit GFP staining restricted to the grafted cells (Figure 5B, right).

To determine the extent of Aβ clearance from the brain due to sNEP delivery, we stained sagittal sections for Aβ immunoreactivity from the medial to the lateral hippocampus. At the graft site, the ipsilateral hippocampus was largely cleared of plaques near the sNEP-producing fibroblasts (Figure 5E, right). There was some reduction in plaque load at the GFP graft (Figure 5F, right), which was likely due to the displacement of plaque-laden tissue by the grafted GFP-expressing fibroblasts. The ipsilateral hippocampus medial to the graft site showed significant reductions in Aβ immunoreactivity in the sNEP-injected mice (compared to the uninjected contralateral hippocampus), whereas no effect was observed in the GFP-injected mice (Figure 5C versus 5D, respectively). Similarly, in the lateral hippocampus distal to the graft, we observed significant reductions in plaque burden due to sNEP expression (Figure 5G versus 5H). Changes in plaque burden were measured in blinded fashion with automated image analysis, and the data expressed as a ratio of the area covered by Aβ plaque on the grafted ipsilateral side versus the control contralateral side. Comparing the ipsilateral/contralateral ratios of the sNEP and GFP groups, significant reductions in plaque burden were observed in the ipsilateral hippocampus from mice engrafted with sNEP-secreting fibroblasts medial to the graft site (34% reduction, *p* < 0.01), within the graft site (72% reduction, *p* < 0.05), and lateral to the graft site (55% reduction, *p* < 0.01) (Figure 5I–5K). Thus, ex vivo gene delivery of a soluble, secreted form of human NEP resulted in substantial and significant reductions in Aβ plaque burden both in the vicinity of the graft and adjacent to the graft site.

### Reduction in Thioflavin-Positive Plaques, but Not Astrocytosis, Following sNEP Cell Engraftment

**Figure 6:**
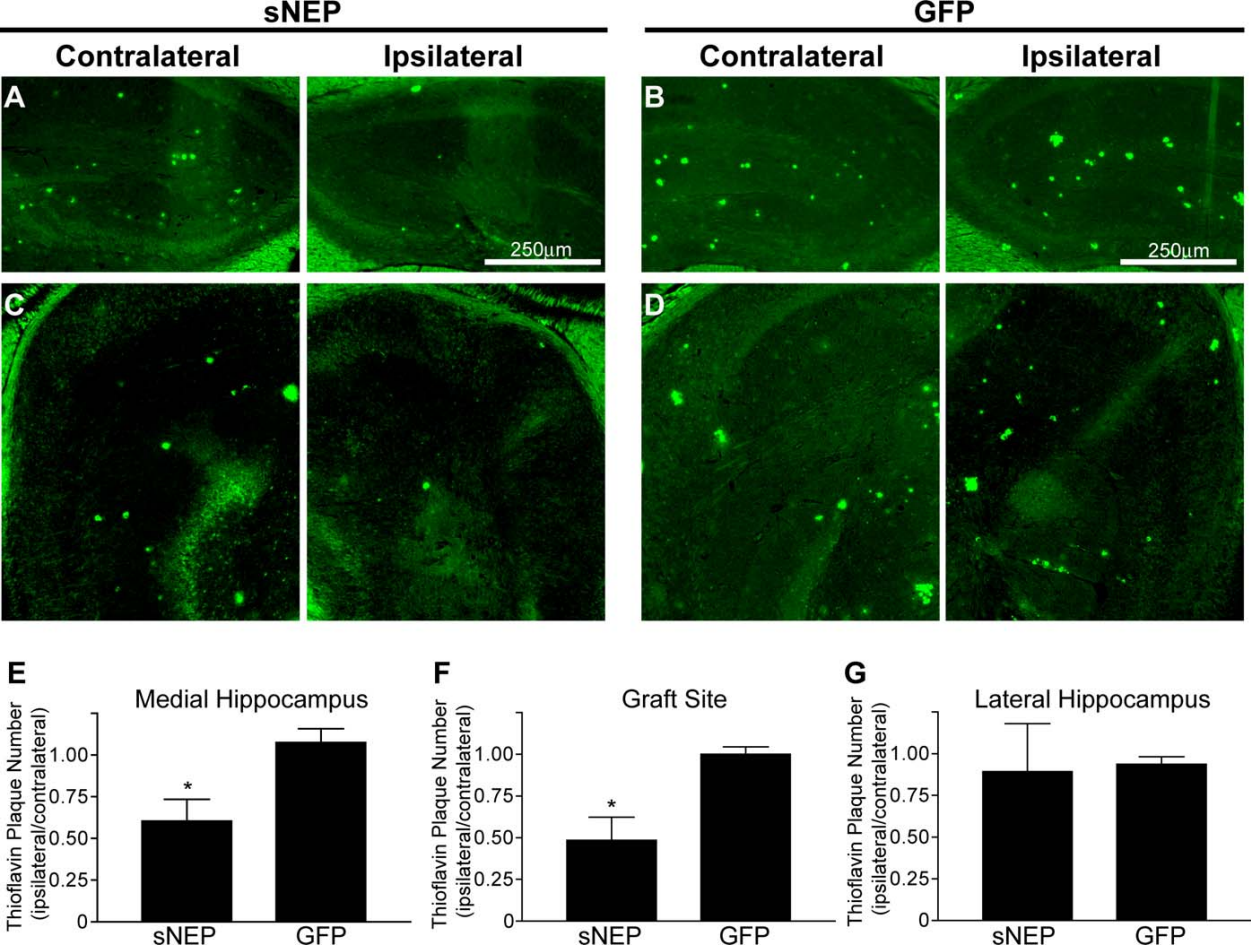
Reduction in Hippocampal Thioflavin Staining Following sNEP Cell Engraftment Brain sections from mice engrafted with cells expressing sNEP or GFP were stained with thioflavin-S to quantify fibrillar plaques. Photomicrographs (A–D) show thioflavin staining at sites medial to the graft (A and B) and at the site of the graft (C and D). Thioflavin-positive plaques were counted, and the ratios of plaques on the ipsilateral versus contralateral hemispheres were calculated for sNEP and GFP groups for the medial hippocampus (E), the graft site (F), and the lateral hippocampus (G). Data represent the mean ± SEM, compared to GFP control: *p = 0.0432 for the medial hippocampus (E), and *p = 0.0337 for the graft site (F). The difference at the lateral hippocampus was not significant.

To determine if the engrafted sNEP was able to clear fibrillar plaques, we examined the number of thioflavin-positive plaques in the ipsilateral and contralateral hippocampus. Sagittal sections throughout the hippocampus were stained with thioflavin-S, and the ratio between the number of plaques on the ipsilateral versus contralateral hippocampus was determined. Comparing the ipsilateral/contralateral ratios from the sNEP and GFP groups, thioflavin-positive plaques were reduced in the medial hippocampus (40% reduction in plaque number, *p* < 0.05; Figure 6A and 6B) and at the graft site (51% reduction in plaque number, *p* < 0.05; Figure 6C and 6D), but was not significantly changed in the lateral hippocampus of the sNEP engrafted brains compared to control (Figure 6E–6G). The lateral hippocampus had few or no thioflavin-positive plaques in either condition and was thus not a reliable reporter of plaque clearance. These data demonstrate that sNEP delivery promotes the clearance of fibrillar, thioflavin-positive plaques from the brain of APP transgenic mice.

**Figure 7:**
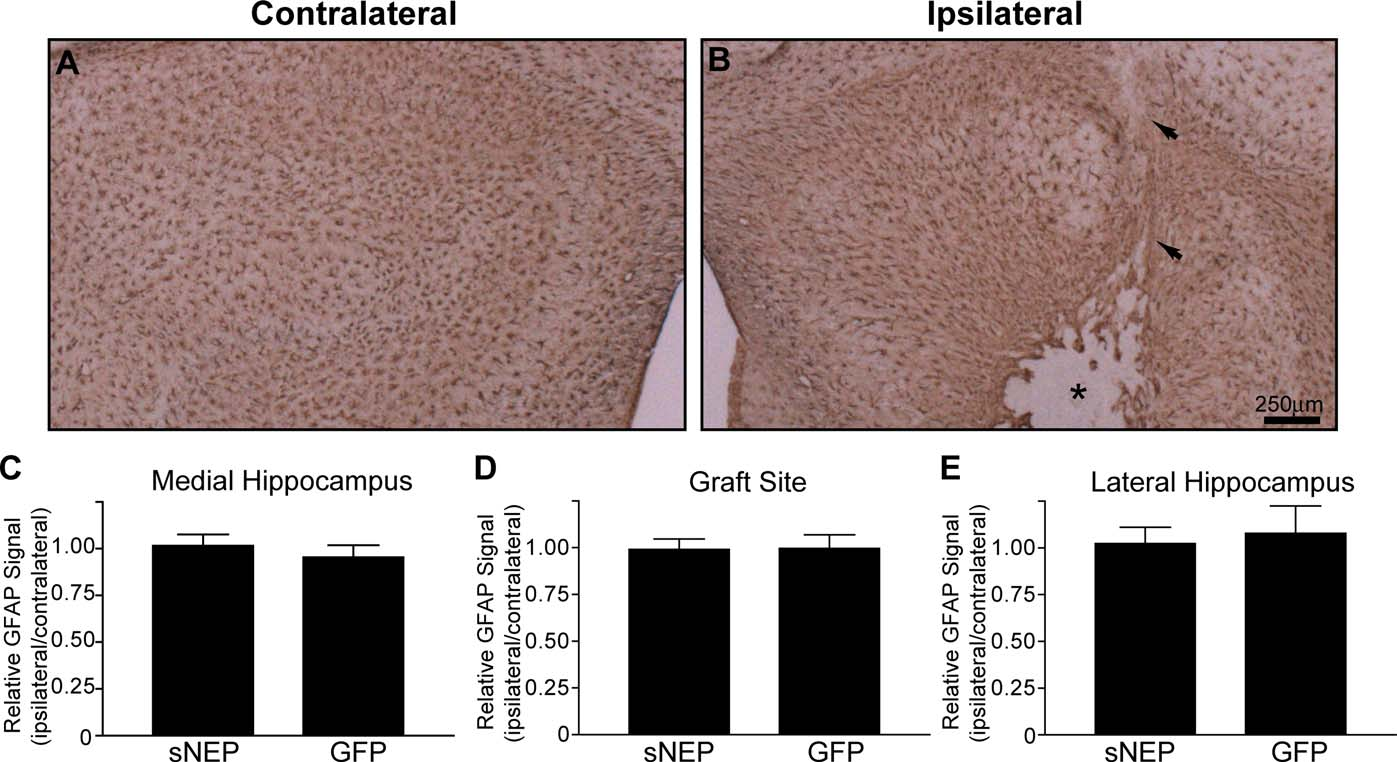
No Change in Overall Astrocytosis following Cell Implantation Brain sections from engrafted mice were stained for GFAP to probe for changes in astrocytosis. (A and B) Compared to the contralateral hemisphere (A), the graft site (B) demonstrated an absence of GFAP staining within the graft and modestly increased astrocyte staining along the border of the graft. The needle track is indicated by arrows and the graft by an asterisk. This staining pattern was observed for both sNEP and GFP conditions (only sNEP is shown). (C–E) The area staining for GFAP on the ipsilateral versus contralateral hippocampus was determined for the medial hippocampus (C), the graft site (D), and the lateral hippocampus (E). Data represent the mean ± SEM.

The activation of astrocyte growth (astrocytosis) is a marker for brain inflammation, and astrocyte hypertrophy and number are increased in AD and may play a role in disease pathogenesis [1]. Transgenic expression of neprilysin has been shown previously to reduce astrocytosis over the lifetime of an APP-transgenic mouse [15], presumably due to reductions in Aβ accumulation. However, because we used a surgical approach to exogenously deliver a soluble form of NEP that clears existing plaques, there could be an inflammatory response associated with plaque removal or with the soluble protease itself. To examine this possibility, we stained sNEP and GFP brains for the astrocyte GFAP. At the site of cell engraftment, few astrocytes were found to have infiltrated the graft, although there was a modest increase in GFAP staining along the edge of the graft and along the needle track (Figure 7A and 7B). However, comparing overall hippocampal GFAP staining on the ipsilateral versus contralateral hemispheres showed that there was no change in astrocytosis due to cell engraftment or sNEP expression in any region of the hippocampus (Figure 7C–7E). Thus, sNEP cell delivery did not promote astrocytosis when measured at 28 d postimplantation. Future experiments examining longer time points of sNEP delivery could show that brain inflammation decreases after Aβ clearance.

## Discussion

Various approaches to lowering Aβ levels within the brain have come to represent the central focus in AD therapeutic development. In this study, we describe the use of ex vivo gene therapy as a viable approach to reducing Aβ levels utilizing a modified Aβ-degrading protease. Replacing the transmembrane domain of the normally membrane-anchored NEP with a signal peptide resulted in the robust secretion of an enzymatically active protease. When syngenic fibroblasts modified to produce sNEP were engrafted into aged APP transgenic mice, the majority of the plaque burden surrounding the graft site was proteolytically cleared. Distal to the graft, in both the medial and lateral hippocampus, significant reductions in plaque burden were also observed as a result of sNEP gene delivery. These results demonstrate the utility of locally elevating the expression of a secreted Aβ-degrading protease as a potential therapeutic approach for AD.

The finding that sNEP was able to reduce plaque burden distal to the graft site may be explained by the diffusion of the sNEP enzyme through the parenchyma, by diffusion of soluble forms of Aβ toward the graft (which may thus be serving as an Aβ sink), or both of these mechanisms. Because of the low sensitivity of available anti-NEP antibodies, we were unable to detect sNEP diffusion farther than ∼800 μm from the graft site, nor could we detect the very low (Figure 1A) endogenous NEP levels expressed by neural tissue [51].

Potential side effects of sNEP expression may arise from the degradation of NEP substrates other than Aβ present in the brain, such as enkephalins. Though studies explicitly searching for such side effects were not performed here, no adverse phenotype was reported in NEP transgenic mice that were protected from Aβ-induced premature death [15], and in this study we observed no enhanced astrocytosis in response to sNEP expression.

A major obstacle to translating the ex vivo methods used in this study into a human disease therapy is the larger dimensions of the human brain, which might require an elevation in Aβ-degrading activity throughout a much broader area. However, our experiment involved just a 28 d exposure to the sNEP-secreting cells, and we found these primary cells to maintain transgene expression in vitro for over a year. One approach to the problem of optimal diffusion of the gene product is to implant the cells where they may have the best access to circulating Aβ, such as in the cerebrospinal fluid or the ventricular wall. Several studies have reported the successful use of encapsulated cell implants that allow for diffusion of therapeutic factors into biological fluids, including cerebrospinal fluid [29,30]. By analogy to the peripheral sink hypothesis for Aβ immunotherapy [52], it may also be feasible and attractive to implant genetically modified cells into peripheral compartments (e.g., the peritoneal cavity or skeletal muscle), where they may substantially increase the Aβ-degrading capacity of the periphery and thus increase the rate of efflux of Aβ across the blood–brain barrier. Moreover, some of the secreted sNEP provided by such a peripheral implant may reach the brain and cerebrospinal fluid to help decrease brain Aβ levels directly. The existence of a subset of hematopoietic cells capable of infiltrating the brain and surrounding plaques has been described [53,54]. Though these cells may not efficiently degrade Aβ themselves, if they could be modified to express an Aβ-degrading protease in a manner similar to the approach we report here, they may provide an anatomically targeted means for local Aβ elimination.

In summary, our findings demonstrate that ex vivo gene delivery of an Aβ-degrading protease rapidly lowers hippocampal Aβ plaque burden in a mouse model of Alzheimer disease. Ex vivo gene delivery and other strategies to elevate Aβ-degrading activity in Alzheimer patients warrant further investigation as potential therapeutic approaches to treat this common and devastating disease.

## Abbreviations

Aβ: amyloid β-protein
ACE: angiotensin-converting enzyme
AD: Alzheimer disease
APP: β-amyloid precursor protein
CHO: Chinese hamster ovary
DAGNPG: 3-dansyl-D-Ala-Gly-p-(nitro)-Phe-Gly
GFAP: glial fibrillary acidic protein
HEK: human embryonic kidney
NEP: neprilysin
SEM: standard error of the mean
sNEP: secreted neprilysin

## References

[pmed-0040262-b001] Selkoe DJ (2001). Alzheimer's disease: Genes, proteins, and therapy. Physiol Rev.

[pmed-0040262-b002] Walsh DM, Klyubin I, Fadeeva JV, Cullen WK, Anwyl R (2002). Naturally secreted oligomers of amyloid beta protein potently inhibit hippocampal long-term potentiation in vivo. Nature.

[pmed-0040262-b003] D'Amore JD, Kajdasz ST, McLellan ME, Bacskai BJ, Stern EA (2003). In vivo multiphoton imaging of a transgenic mouse model of Alzheimer disease reveals marked thioflavine-S-associated alterations in neurite trajectories. J Neuropathol Exp Neurol.

[pmed-0040262-b004] Lewis J, Dickson DW, Lin WL, Chisholm L, Corral A (2001). Enhanced neurofibrillary degeneration in transgenic mice expressing mutant tau and APP. Science.

[pmed-0040262-b005] Oddo S, Billings L, Kesslak JP, Cribbs DH, LaFerla FM (2004). Abeta immunotherapy leads to clearance of early, but not late, hyperphosphorylated tau aggregates via the proteasome. Neuron.

[pmed-0040262-b006] Siemers ER, Quinn JF, Kaye J, Farlow MR, Porsteinsson A (2006). Effects of a gamma-secretase inhibitor in a randomized study of patients with Alzheimer disease. Neurology.

[pmed-0040262-b007] Gilman S, Koller M, Black RS, Jenkins L, Griffith SG (2005). Clinical effects of Abeta immunization (AN1792) in patients with AD in an interrupted trial. Neurology.

[pmed-0040262-b008] Hock C, Konietzko U, Streffer JR, Tracy J, Signorell A (2003). Antibodies against beta-amyloid slow cognitive decline in Alzheimer's disease. Neuron.

[pmed-0040262-b009] Iwata N, Tsubuki S, Takaki Y, Shirotani K, Lu B (2001). Metabolic regulation of brain Abeta by neprilysin. Science.

[pmed-0040262-b010] Farris W, Mansourian S, Chang Y, Lindsley L, Eckman EA (2003). Insulin-degrading enzyme regulates the levels of insulin, amyloid beta -protein, and the beta -amyloid precursor protein intracellular domain in vivo. Proc Natl Acad Sci U S A.

[pmed-0040262-b011] Miller BC, Eckman EA, Sambamurti K, Dobbs N, Chow KM (2003). Amyloid-beta peptide levels in brain are inversely correlated with insulysin activity levels in vivo. Proc Natl Acad Sci U S A.

[pmed-0040262-b012] Eckman EA, Watson M, Marlow L, Sambamurti K, Eckman CB (2003). Alzheimer's disease beta-amyloid peptide is increased in mice deficient in endothelin-converting enzyme. J Biol Chem.

[pmed-0040262-b013] Melchor JP, Pawlak R, Strickland S (2003). The tissue plasminogen activator-plasminogen proteolytic cascade accelerates amyloid-beta (Abeta) degradation and inhibits Abeta-induced neurodegeneration. J Neurosci.

[pmed-0040262-b014] Mueller-Steiner S, Zhou Y, Arai H, Roberson ED, Sun B (2006). Antiamyloidogenic and neuroprotective functions of cathepsin B: Implications for Alzheimer's disease. Neuron.

[pmed-0040262-b015] Leissring MA, Farris W, Chang AY, Walsh DM, Wu X (2003). Enhanced proteolysis of beta-amyloid in APP transgenic mice prevents plaque formation, secondary pathology, and premature death. Neuron.

[pmed-0040262-b016] Iwata N, Mizukami H, Shirotani K, Takaki Y, Muramatsu S (2004). Presynaptic localization of neprilysin contributes to efficient clearance of amyloid-beta peptide in mouse brain. J Neurosci.

[pmed-0040262-b017] Marr RA, Rockenstein E, Mukherjee A, Kindy MS, Hersh LB (2003). Neprilysin gene transfer reduces human amyloid pathology in transgenic mice. J Neurosci.

[pmed-0040262-b018] Dwarki VJ, Belloni P, Nijjar T, Smith J, Couto L (1995). Gene therapy for hemophilia A: Production of therapeutic levels of human factor VIII in vivo in mice. Proc Natl Acad Sci U S A.

[pmed-0040262-b019] Uteza Y, Rouillot JS, Kobetz A, Marchant D, Pecqueur S (1999). Intravitreous transplantation of encapsulated fibroblasts secreting the human fibroblast growth factor 2 delays photoreceptor cell degeneration in Royal College of Surgeons rats. Proc Natl Acad Sci U S A.

[pmed-0040262-b020] Peron JM, Bureau C, Gourdy P, Lulka H, Souque A (2007). Treatment of experimental murine pancreatic peritoneal carcinomatosis with fibroblasts genetically modified to express IL12: A role for peritoneal innate immunity. Gut.

[pmed-0040262-b021] Blesch A, Tuszynski MH (2003). Cellular GDNF delivery promotes growth of motor and dorsal column sensory axons after partial and complete spinal cord transections and induces remyelination. J Comp Neurol.

[pmed-0040262-b022] Lattanzi L, Salvatori G, Coletta M, Sonnino C, Cusella De Angelis MG (1998). High efficiency myogenic conversion of human fibroblasts by adenoviral vector-mediated MyoD gene transfer. An alternative strategy for ex vivo gene therapy of primary myopathies. J Clin Invest.

[pmed-0040262-b023] Ishii S, Koyama H, Miyata T, Nishikage S, Hamada H (2004). Appropriate control of ex vivo gene therapy delivering basic fibroblast growth factor promotes successful and safe development of collateral vessels in rabbit model of hind limb ischemia. J Vasc Surg.

[pmed-0040262-b024] Frim DM, Uhler TA, Galpern WR, Beal MF, Breakefield XO (1994). Implanted fibroblasts genetically engineered to produce brain-derived neurotrophic factor prevent 1-methyl-4-phenylpyridinium toxicity to dopaminergic neurons in the rat. Proc Natl Acad Sci U S A.

[pmed-0040262-b025] Emerich DF, Winn SR, Hantraye PM, Peschanski M, Chen EY (1997). Protective effect of encapsulated cells producing neurotrophic factor CNTF in a monkey model of Huntington's disease. Nature.

[pmed-0040262-b026] Sagot Y, Tan SA, Baetge E, Schmalbruch H, Kato AC (1995). Polymer encapsulated cell lines genetically engineered to release ciliary neurotrophic factor can slow down progressive motor neuronopathy in the mouse. Eur J Neurosci.

[pmed-0040262-b027] Tuszynski MH, Smith DE, Roberts J, McKay H, Mufson E (1998). Targeted intraparenchymal delivery of human NGF by gene transfer to the primate basal forebrain for 3 months does not accelerate beta-amyloid plaque deposition. Exp Neurol.

[pmed-0040262-b028] Tuszynski MH, Thal L, Pay M, Salmon DP, U HS (2005). A phase 1 clinical trial of nerve growth factor gene therapy for Alzheimer disease. Nat Med.

[pmed-0040262-b029] Aebischer P, Schluep M, Deglon N, Joseph JM, Hirt L (1996). Intrathecal delivery of CNTF using encapsulated genetically modified xenogeneic cells in amyotrophic lateral sclerosis patients. Nat Med.

[pmed-0040262-b030] Sieving PA, Caruso RC, Tao W, Coleman HR, Thompson DJ (2006). Ciliary neurotrophic factor (CNTF) for human retinal degeneration: phase I trial of CNTF delivered by encapsulated cell intraocular implants. Proc Natl Acad Sci U S A.

[pmed-0040262-b031] Simons JW, Mikhak B, Chang JF, DeMarzo AM, Carducci MA (1999). Induction of immunity to prostate cancer antigens: Results of a clinical trial of vaccination with irradiated autologous prostate tumor cells engineered to secrete granulocyte-macrophage colony-stimulating factor using ex vivo gene transfer. Cancer Res.

[pmed-0040262-b032] Hama E, Shirotani K, Iwata N, Saido TC (2004). Effects of neprilysin chimeric proteins targeted to subcellular compartments on amyloid beta peptide clearance in primary neurons. J Biol Chem.

[pmed-0040262-b033] Russo R, Borghi R, Markesbery W, Tabaton M, Piccini A (2005). Neprylisin decreases uniformly in Alzheimer's disease and in normal aging. FEBS Lett.

[pmed-0040262-b034] Yasojima K, Akiyama H, McGeer EG, McGeer PL (2001). Reduced neprilysin in high plaque areas of Alzheimer brain: A possible relationship to deficient degradation of beta-amyloid peptide. Neurosci Lett.

[pmed-0040262-b035] Hemming ML, Selkoe DJ (2005). Amyloid beta-protein is degraded by cellular angiotensin-converting enzyme (ACE) and elevated by an ACE inhibitor. J Biol Chem.

[pmed-0040262-b036] Citron M, Oltersdorf T, Haass C, McConlogue L, Hung AY (1992). Mutation of the beta-amyloid precursor protein in familial Alzheimer's disease increases beta-protein production. Nature.

[pmed-0040262-b037] Podlisny MB, Ostaszewski BL, Squazzo SL, Koo EH, Rydell RE (1995). Aggregation of secreted amyloid beta-protein into sodium dodecyl sulfate-stable oligomers in cell culture. J Biol Chem.

[pmed-0040262-b038] Dull T, Zufferey R, Kelly M, Mandel RJ, Nguyen M (1998). A third-generation lentivirus vector with a conditional packaging system. J Virol.

[pmed-0040262-b039] Hemming ML, Selkoe DJ, Farris W (2007). Effects of prolonged angiotensin-converting enzyme inhibitor treatment on amyloid β-protein metabolism in mouse models of Alzheimer disease. Neurobiol Dis.

[pmed-0040262-b040] Florentin D, Sassi A, Roques BP (1984). A highly sensitive fluorometric assay for “enkephalinase,” a neutral metalloendopeptidase that releases tyrosine-glycine-glycine from enkephalins. Anal Biochem.

[pmed-0040262-b041] Johnson-Wood K, Lee M, Motter R, Hu K, Gordon G (1997). Amyloid precursor protein processing and A beta42 deposition in a transgenic mouse model of Alzheimer disease. Proc Natl Acad Sci U S A.

[pmed-0040262-b042] Mucke L, Masliah E, Yu GQ, Mallory M, Rockenstein EM (2000). High-level neuronal expression of abeta 1–42 in wild-type human amyloid protein precursor transgenic mice: Synaptotoxicity without plaque formation. J Neurosci.

[pmed-0040262-b043] Abramoff MD, Magelhaes PJ, Ram SJ (2004). Image processing with image J. Biophotonics International.

[pmed-0040262-b044] Iwata N, Tsubuki S, Takaki Y, Watanabe K, Sekiguchi M (2000). Identification of the major Abeta1–42-degrading catabolic pathway in brain parenchyma: Suppression leads to biochemical and pathological deposition. Nat Med.

[pmed-0040262-b045] Huang SM, Mouri A, Kokubo H, Nakajima R, Suemoto T (2006). Neprilysin-sensitive synapse-associated amyloid-beta peptide oligomers impair neuronal plasticity and cognitive function. J Biol Chem.

[pmed-0040262-b046] Lafrance MH, Vezina C, Wang Q, Boileau G, Crine P (1994). Role of glycosylation in transport and enzymic activity of neutral endopeptidase-24.11. Biochem J.

[pmed-0040262-b047] Tuszynski MH, Senut MC, Ray J, Roberts J (1994). Somatic gene transfer to the adult primate central nervous system: In vitro and in vivo characterization of cells genetically modified to secrete nerve growth factor. Neurobiol Dis.

[pmed-0040262-b048] Wen J, Vargas AG, Ofosu FA, Hortelano G (2006). Sustained and therapeutic levels of human factor IX in hemophilia B mice implanted with microcapsules: Key role of encapsulated cells. J Gene Med.

[pmed-0040262-b049] Tobias CA, Shumsky JS, Shibata M, Tuszynski MH, Fischer I (2003). Delayed grafting of BDNF and NT-3 producing fibroblasts into the injured spinal cord stimulates sprouting, partially rescues axotomized red nucleus neurons from loss and atrophy, and provides limited regeneration. Exp Neurol.

[pmed-0040262-b050] Tuszynski MH (2002). Growth-factor gene therapy for neurodegenerative disorders. Lancet Neurol.

[pmed-0040262-b051] Li C, Booze RM, Hersh LB (1995). Tissue-specific expression of rat neutral endopeptidase (neprilysin) mRNAs. J Biol Chem.

[pmed-0040262-b052] Citron M (2004). Strategies for disease modification in Alzheimer's disease. Nat Rev Neurosci.

[pmed-0040262-b053] Simard AR, Soulet D, Gowing G, Julien JP, Rivest S (2006). Bone marrow-derived microglia play a critical role in restricting senile plaque formation in Alzheimer's disease. Neuron.

[pmed-0040262-b054] Stalder AK, Ermini F, Bondolfi L, Krenger W, Burbach GJ (2005). Invasion of hematopoietic cells into the brain of amyloid precursor protein transgenic mice. J Neurosci.

